# Allergen Sensitization and Asthma Outcomes among World Trade Center Rescue and Recovery Workers

**DOI:** 10.3390/ijerph16050737

**Published:** 2019-03-01

**Authors:** Belen Rojano, Erin West, Emily Ferdermann, Steven Markowitz, Denise Harrison, Laura Crowley, Paula Busse, Alex D. Federman, Juan P. Wisnivesky

**Affiliations:** 1 Division of General Internal Medicine, Department of Medicine, Icahn School of Medicine at Mount Sinai, New York, NY 10029, USA; belen.rojanobroz@mountsinai.org (B.R.); krystallyn@gmail.com (E.W.); emily.federmann@mountsinai.org (E.F.); alex.federman@mountsinai.org (A.D.F.); 2 Barry Commoner Center for Health and the Environment, Queens College, City University of New York, Queens, New York, NY 11367, USA; steven.markowitz@qc.cuny.edu; 3 Department of Medicine, New York University School of Medicine, Bellevue Hospital Center, New York, NY 10016, USA; denise.harrison@nyulangone.org; 4 Division of Occupational and Environmental Medicine, Department of Environmental Medicine and Public Health, Icahn School of Medicine at Mount Sinai, New York, NY 10029, USA; laura.crowley@mssm.edu; 5 Division of Allergy and Immunology, Department of Medicine, Icahn School of Medicine at Mount Sinai, New York, NY 10029, USA; paula.busse@mssm.edu; 6 Division of Pulmonary, Critical Care, and Sleep Medicine, Department of Medicine, Icahn School of Medicine at Mount Sinai, New York, NY 10029, USA

**Keywords:** World Trade Center, indoor allergens sensitization, asthma quality of life, asthma control, asthma outcomes, mini asthma quality of life questionnaire, asthma morbidity, WTC-related asthma, immunoglobulin E, allergen exposure

## Abstract

A large number of World Trade Center (WTC) rescue and recovery workers are affected by asthma. While physical and mental health comorbidities have been associated with poor asthma control in this population, the potential role of allergen sensitization is unknown. This study examined the association of indoor sensitization and exposure as a risk factor for increased asthma morbidity in WTC workers. We used data from a prospective cohort of 331 WTC workers with asthma. Sensitization to indoor allergens was assessed by measurement of antigen-specific serum immunoglobulin E (IgE) levels. We used validated tools to evaluate the exposure to indoor allergens. Asthma morbidity outcomes included level of control (Asthma Control Questionnaire, ACQ), quality of life (Asthma Quality of Life Questionnaire, AQLQ) and acute resource utilization. The prevalence of sensitization to cat, dog, mouse, dust mite, cockroach, and mold allergens were 33%, 21%, 17%, 40%, 17%, and 17%, respectively. Unadjusted and regression analyses showed no significant relationship between sensitization and increased asthma morbidity (*p* > 0.05 for all comparisons), except for sensitization to Aspergillus Fumigatus, cat and mouse epithelium, which were associated with decreased morbidity.

## 1. Introduction

Multiple studies have documented high rates of asthma prevalence (approximately 30% cumulative incidence, 9 years after exposure) among World Trade Center (WTC) rescue and recovery workers [1,2]. Recent studies have also demonstrated that many WTC-exposed individuals suffer from substantial asthma morbidity, including poor disease control and a relatively high number of emergency room visits and hospitalizations [1]. The reasons for the poor outcomes observed among WTC workers are likely multifactorial and partially explained by an increased prevalence of comorbidities, such as gastroesophageal reflux (GERD) and chronic sinusitis, which are known to worsen asthma [1,3]. Similarly, post-traumatic stress disorder (PTSD), which has been described in up to 30% of WTC workers, is strongly associated with increased asthma morbidity [4].

Allergic sensitization is also associated with increased asthma morbidity, particularly in inner-city children [5,6]. Moreover, environmental remediation strategies to reduce indoor exposures have been shown to improve asthma control in this population [7,8]. The results of studies assessing the role of allergic sensitization in adult asthmatics are mixed [9,10,11]. Nonetheless, anti-immunoglobulin E (IgE) therapy is effective for antigen-sensitized patients and trigger avoidance is currently recommended as a major component of asthma self-management by the most recent national asthma guidelines [12]. The role sensitization and exposure to allergens on asthma outcomes in WTC workers has not been previously explored.

In this study, we determined the rates of sensitization to indoor allergens in a cohort of WTC workers with asthma, and assessed the relationship to asthma control and acute resource utilization.

## 2. Materials and Methods

### 2.1. Study Population

The study was conducted using data from a cohort of WTC workers with a physician diagnosis of asthma. Study participants were recruited between December 2012 and July 2016, from WTC workers who were followed by the Mount Sinai Hospital, North Shore-Long Island Jewish Health System/Queens College, and the New York University School of Medicine WTC Health Program. Criteria for recruitment into this program have been previously published [4,13] and include individuals who have volunteered or worked in the lower Manhattan, barge-loading piers or Staten Island landfill, and workers from the Port Authority Trans Hudson Corporation who were engaged in cleaning and personnel of the Office of the Chief Medical Examiner who processed human remains. Lower Manhattan residents, schoolchildren, building occupants, and passers-by were not included in this registry. Members of the Fire Department of the City of New York (FDNY) who are followed in a parallel program were also not included in the present study.

The current study was limited to patients with physician-diagnosed asthma who spoke English or Spanish and were ≥18 years of age at the time of enrollment. We excluded WTC workers with a prior diagnosis of chronic obstructive lung disease (COPD) and those who had history of >15 pack-years of smoking, due to the possibility of undiagnosed COPD. We also excluded workers with other chronic respiratory illnesses. Signed consent was obtained from all participants; the Institutional Review Boards of the Icahn School of Medicine at Mount Sinai, Queens College and New York University School of Medicine approved this study.

### 2.2. Study Variables

Study participants underwent an in-person standardized interview in English or Spanish to collect sociodemographic information and data regarding asthma history, including onset in relation to WTC exposure and medication regimen. We obtained information about physician diagnosis of GERD, allergic rhinitis, and chronic sinusitis, as well as other comorbidities. In order to diagnose the presence of mental health conditions (PTSD, panic disorder and depression) patients underwent a structured clinical psychiatric interview (SCID) [14]. According to criteria published in previous studies, participants were assigned to one of four different groups depending on the level of WTC exposure: Low, intermediate, high, and very high [1].

### 2.3. Allergic Sensitization and Exposure Assessments

Sensitization to indoor allergens was assessed using serum IgE levels in peripheral blood; a level >0.35 kU/L was considered indicative of sensitization [12,15]. The allergens included were, cat epithelium and dander, dog, mouse epithelium, house dust mites (*Dermatophagoides Farinae* and *Dermatophagoides Pteronyssinus*), cockroaches (*Blatella Germanica* or *Periplaneta Americana*), and molds (*Alternaria Alternata* and *Aspergillus Fumigatus*). Serum IgE levels were determined using the Thermo Fisher Scientific Analyzer Phadia™ 1000^®^ (Phadia AB, Uppsala, Sweden). Home environmental exposures were ascertained using survey questions previously validated against findings from home inspections [16,17,18]. The survey included questions about the presence of pets at home, visible molds, mildew, wet spots, and/or cockroaches. In addition, study participants were asked about the presence of dust at home, home cleaning behaviors, and if the participants kept windows closed during the allergy seasons.

### 2.4. Outcomes

We used the Asthma Control Questionnaire (ACQ) to assess the level of asthma control [19]. The ACQ is a validated tool available in English and Spanish and has been extensively used in clinical practice and research [20]. Higher ACQ scores indicate worse asthma control and a change of >0.5 units is considered clinically significant [19]. The impact of allergic sensitization on quality of life was assessed with the Mini Asthma Quality of Life Questionnaire (AQLQ) [21]. This validated tool includes 15 questions in four domains (symptoms, environment, emotions and activities) and has good reliability and responsiveness [21,22]. A higher score on the AQLQ indicates better quality of life related to asthma [21]. We also collected information about asthma-related resource utilization (visits to emergency department, hospitalizations, use of oral corticosteroids) in the previous year.

### 2.5. Statistical Analysis

The means with standard deviations and percentages with 95% confidence intervals (CI) were used to describe the baseline characteristics of the study participants. We used the *t*-test to compare ACQ and AQLQ scores of WTC workers sensitized versus those not sensitized to each specific indoor allergen. The chi-square test was used to compare acute resource utilization according to sensitization status. The adjusted association between sensitization status, ACQ, and AQLQ scores over time was assessed using linear regression to control for sociodemographic characteristics, asthma history, asthma onset in relation to 9/11 exposure, asthma regimen, WTC exposure level, and comorbidities. The potential relationship of acute resource utilization with sensitization to each allergen was evaluated using logistic regression analysis.

Power calculations showed that a total of approximately 220 patients were required for the study to have 80% power to detect a clinically significance difference of ≥0.5 units in ACQ and AQLQ scores among patients sensitized versus not sensitized to each indoor allergen. All statistical tests were performed with SAS statistical software (SAS Institute, Cary, NC, USA) using 2-tailed tests.

## 3. Results

### 3.1. Participant Characteristics

Overall, 373 WTC workers with asthma were enrolled in the study; of these, 42 lacked results for specific IgE and were excluded from these analyses. The mean (SD) age of study participants was 52.7 (8) years; 73% were male; 35% white, 13% Black, and 43% Hispanic (Table 1). Most WTC workers (72%) reported asthma onset after WTC exposure and 66% were prescribed an asthma controller medication. Among the cohort, the frequency of patients with well-controlled, uncontrolled, and very poorly controlled asthma was 27%, 26%, and 47%, respectively; almost half (49%) reported poor quality of life. In terms of resource utilization, 20% of WTC workers had a hospitalization or an ER visit related to their asthma in the previous 12 months, and 27% had received an oral corticosteroid burst for an exacerbation.

Overall, 56% (95% CI: 51–62%) of WTC workers were sensitized to at least one indoor allergen (Table 2). The frequency of sensitization to cat, dog, mouse epithelium, *Dermatophagoides Farinae*, *Dermatophagoides Pteronyssines*, cockroach, *Alternaria Tenuis*, and *Aspergillus Fumigatus* was 33% (95% CI: 28–38%), 21% (95% CI: 17–26%), 17% (95% CI: 13–22%), 34% (95% CI: 30–40%), 32% (95% CI: 26–37%), 17% (95% CI: 13–21%), 11% (95% CI: 7–14%), and 10% (95% CI: 6–15%), respectively. Overall, 50% of participants had cats, dogs, or birds at home, 17% reported presence of mice/rats at home, 24% had observed cockroaches, 20% observed wet spots, 25% had mold/mildew at their home, and only 34% kept windows closed during the allergy season.

### 3.2. Unadjusted Associations between Sensitization and Asthma Morbidity

Unadjusted analyses showed no significant association between sensitization to most indoor allergen and ACQ and AQLQ scores (*p* > 0.05 for all comparisons) (Table 3). Only sensitization to Aspergillus Fumigatus (mean difference: −0.63; 95% CI: −1.17 to −0.09) was associated with a lower ACQ score. Similarly, sensitization to at least one allergen (mean difference: 0.29; 95% CI: 0.01 to 0.56), and sensitization to cat allergens (mean difference: 0.36; 95% CI: 0.08 to 0.65) were associated with higher AQLQ scores. In addition, the use of oral corticosteroids and acute resource utilization were not significantly associated with sensitization status to most allergens (*p* > 0.05 for all comparisons). However, sensitization to mouse epithelium (OR: 0.39; 95% CI: 0.18–0.86) was associated with a decreased use of oral corticoids in the year before enrollment. We also found that acute asthma-related resource utilization was lower in patients sensitized to at least one allergen (OR: 0.54; 95% CI: 0.29–0.99). Unadjusted analyses also showed no significant interaction between allergen sensitization and WTC exposure category (*p* > 0.05 for all comparisons), suggesting the effect of sensitization was not different according to the level of exposure at the WTC site.

### 3.3. Adjusted Associations between Indoor Sensitization and Asthma Control

Adjusted analyses also showed no statistically significant association between the sensitization to most allergens and ACQ and AQLQ scores, use of oral steroids, and previous utilization of health care services (*p* > 0.05 for all comparisons) (Table 4). However, sensitization to at least one indoor allergen (mean difference: 0.27; 95% CI: 0.03 to 0.50) or Dermatophagoides Pteronyssines (mean difference: 0.29; 95% CI: 0.03 to 0.55) were significantly associated with higher AQLQ scores. Finally, sensitization to at least one allergen (OR: 0.41; 95% CI: 0.20–0.85), and cockroach (OR: 0.27; 95% CI: 0.08–0.91) were significantly associated with lower rates of acute asthma-related resource utilization.

## 4. Discussion

Asthma is a common WTC-related condition that is frequently poorly controlled and associated with substantial healthcare resource utilization [1]. In this study, we found that a large percentage of WTC workers are sensitized to common indoor allergens, similarly to the general population [23]. However, sensitization to most of the indoor allergens evaluated was not significantly associated with increased asthma morbidity or acute resource utilization, even among WTC workers exposed to these allergens. These results suggest that efforts to better control WTC-related asthma should focus on other established risk factors, such as other medical co-morbidities, PTSD, or low adherence to controller medication, and other effective self-management behaviors [1,2,4].

There is strong epidemiologic evidence linking allergen sensitization to increased asthma morbidity in inner-city children [5,6,24,25]. Moreover, the effectiveness of home-based environmental remediation and multi-trigger interventions in decreasing allergen exposure and reducing asthma morbidity is well-established [26,27]. Several studies have also evaluated the potential impact of allergic sensitization on asthma outcomes in adults. Specific allergens, in particular cockroach [5] and *Alternaria* [28], have been associated with more severe asthma; asthma deaths have been linked to high fungal spore counts in the environment [29]. Similarly, a study of 140 inner-city women with asthma showed a statistically significant association between increased asthma morbidity and exposure to cats among sensitized patients [30]. A study of elderly inner-city asthmatics showed that patients with high levels of cockroach-specific IgE had worse disease control and more severe airflow obstruction [10]. Similarly, a study of 5845 adults with asthma showed higher exacerbation rates in those sensitized to cat or dogs that had these pets at home [31]. Conversely, a study of 245 inner-city adults with persistent asthma showed no significant relationship between indoor allergen sensitization and asthma outcomes [9]. The European Respiratory Health Survey showed no association between the levels of cat allergen and/or sensitization status with asthma symptoms [32]. Our study extends these findings by showing a lack of association between sensitization and asthma morbidity in WTC workers.

Several factors may explain our findings. WTC-related asthma may be associated with a higher prevalence of airway neutrophilia [33]. Asthma is characterized by airway inflammation [34], characterized by an eosinophil-dominated pattern, especially in patients who have allergen-induced symptoms and an underlying T2 inflammatory profile. However, asthma is a heterogeneous disease and there is increased evidence that other mechanisms, such as a neutrophil-mediated inflammation, less often associated with atopy, could lead to enhanced bronchial reactivity and airflow obstruction in some patients [35]. Th-17 cells appear to play a key role in the pathophysiology of neutrophilic asthma, a disease often associated with worse control and resistance to inhaled corticosteroids [34,36,37,38,39,40,41,42,43]. Thus, if a larger proportion of WTC workers have neutrophil predominant asthma, it is expected that sensitization plays a decreased role in the underlying pathophysiology of this asthma phenotype, and hence its control. Further understanding about the most common patterns of airway inflammation in WTC workers with asthma is important for personalizing their management.

Many WTC workers have physical and/or mental health comorbidities known to increase asthma morbidity. These include GERD, chronic sinusitis, and rhinitis, as well as, PTSD, and major depression [1,3,4]. Thus, these conditions may be more important determinants of the level of asthma control in WTC workers, rather than the potential impact of allergic sensitization. While prior studies have showed that there is a strong negative impact of allergic sensitization among inner-city children with asthma, our cohort was limited to adults, who spent less time at home and therefore are less exposed to the home indoor environment [44].

Given the relatively high rate of allergen sensitization in our population of WTC workers with asthma, avoidance of environmental triggers could still contribute to improved control in some of these patients, particularly those with features of eosinophilic disease. However, our findings suggest that healthcare providers should also focus on other important asthma triggers in this population, such as other medical comorbidities and occupational exposures.

Our study has some limitations that should be mentioned. Our data were collected between 11 and 15 years of the WTC exposure, and therefore our findings represent a narrow window in time. Despite enrolling a relatively large number of WTC workers, our cohort did not represent all populations exposed to WTC disaster, such as firefighters, local residents, and passersby. Thus, our results may not be generalizable to these groups. Our study did not include a non-WTC asthma control group to assess for potential differences in the role and the impact of allergen sensitization. WTC rescue and recovery workers are a unique population given their exposures at the WTC site, range of occupations and associated exposures prior and after 9/11, healthy worker effect, and a high prevalence of physical and mental health comorbidities, many of which (e.g., GERD and PTSD) are associated with worse asthma control. Thus, it is very difficult to identify a control group to perform robust comparisons of the relationship between allergic sensitization and asthma morbidity in WTC versus no-WTC populations. However, in a prior study of non-WTC asthma patients recruited from the same institution [9], we found similar rates of sensitization to cats (41%), mouse epithelium (14%), dust mites (43%), molds (21%) than those reported in our WTC workers cohort. The prevalence of sensitization to cockroach was higher in the non-WTC asthma cohort (60% vs. 17%) that could be related to higher levels of cockroach exposure in this inner-city population and may explain some of the differences in our results. In this non-WTC population, adjusted analyses showed that allergy to cockroach, dust mites, cat, mouse, or molds were not associated with increased resource utilization or worse asthma outcomes. Some exceptions were the presence of dust mite sensitization that was related to increased use of oral steroids in the year before enrollment, mouse sensitization that was related with an increased number of hospitalizations, and cat sensitization that showed decreased number to ED visits during the follow up period. Similarly, our adjusted analyses showed significant associations between sensitization to some allergens (*Dermatophagoides Pteronyssines* and cockroach) with markers of decreased asthma morbidity. We used objective measures of allergen sensitization and validated cut-offs, however, we did not collect home samples to assess the actual levels of environmental exposure. Although self-reported measures may be subject to bias, we used validated items that have been correlated to direct home environmental assessments [16,21,22]. Similarly, we used validated instruments to assess asthma control and quality of life in study participants. We did not evaluate IgE sensitization to outdoor allergens, as they play a limited role in asthma exacerbations [45]. We found that sensitization to some allergens was associated with improved asthma control. However, these relationships were inconsistent across different asthma outcomes for specific allergens, and thus, more likely related to random sampling. Conversely, our study was not powered to find small differences in asthma control and quality of life among sensitized WTC workers; however, our sample was sufficient for identifying clinically meaningful differences in these outcomes. Finally, we did not assess the potential impact of outdoor allergens on asthma morbidity levels of WTC workers.

## 5. Conclusions

In summary, we found no significant associations between indoor allergen sensitization and exposure to asthma morbidity in WTC workers. Given the high rates of the absence of asthma control and decreased quality of life in this population, our findings can guide providers in their management strategies for WTC workers with asthma.

## Figures and Tables

**Table 1:** Baseline characteristics of World Trade Center rescue and recovery workers with asthma.

Characteristic	Value
Age, years, mean (SD)	52.7 (8)
Male, No. (%)	240 (73)
Race/Ethnicity, No. (%)	
White	115 (35)
Black	44 (13)
Hispanic	142 (43)
Other	28 (8)
Refused/Unknown	2 (1)
Education, No. (%)	
Did not graduate high school	30 (9)
High School or GED	52 (16)
Some College	135 (41)
College Graduate or More Advanced Degree	114 (34)
Monthly Income < $3,000, No. (%)	183 (55)
Occupation, No. (%)	
Employed Full Time	141 (43)
Employed Part Time	35 (10)
Unemployed	19 (6)
On Disability	40 (12)
Retired	72 (22)
Not Working/Student/Other	24 (7)
Smoking Status No. (%)	
Current/former Smoker	101 (31)
Never Smoked	220 (66)
Refused/Unknown	10 (3)
WTC Exposure No. (%)	
Low	51 (15)
Intermediate	138 (42)
High	116 (35)
Very High	26 (8)
Asthma Onset Post 9/11, No. (%)	242 (72)
Hospitalization/Emergency Room Visit for Asthma in the Past Year, No. (%)	64 (20)
Oral Corticosteroid Use in Past 12 Months No. (%)	88 (27)
Asthma Control Level, No. (%)	
Well Controlled	91 (27)
Uncontrolled	86 (26)
Very Poorly Controlled	154 (47)
Asthma-related Quality of Life, No. (%)	
Good	172 (52)
Poor	159 (48)
On Asthma Controller Medication, No. (%)	218 (66)
Comorbidities No. (%)	
Gastric Esophageal Reflux Disorder	222 (67)
Sinusitis	207 (63)
Major Depression	88 (27)
Posttraumatic Stress Disorder	81 (24)

SD: standard deviation. No.: Number. WTC: World Trade Center.

**Table 2:** Sensitization and exposure to indoor allergens among World Trade Center rescue and recovery workers with asthma.

Allergen	Number	Percentage (95% CI)
At least One Indoor Allergens	186	56 (51–62)
Cat Dander	110	33 (28–38)
Dog Dander	63	21 (17–26)
Mouse Epithelium	57	17 (13–22)
*Dermatophagoides Farinae*	115	34 (29–40)
*Dermatophagoides Pteronyssines*	105	32 (26–37)
Cockroach	57	17 (13–21)
*Alternaria Tenuis*	35	11 (7–14)
*Aspergillus Fumigatus*	20	10 (6–15)
**Home Exposures**		
Cats, dogs or birds living in home	165	50 (44–55)
Mice or rats	58	17 (13–22)
Cockroaches	79	24 (19–29)
Wet spots on walls, wallpaper, ceilings or carpets	68	20 (16–25)
Mold or mildew growing on surfaces	83	25 (20–30)
Keeps windows closed during allergen season	110	34 (29–40)

CI: confidence interval.

**Table 3:** Unadjusted associations between sensitization to indoor allergens and asthma morbidity in World Trade Center rescue and recovery workers.

Exposure	Mean ACQ Difference, 95% CI	Mean AQLQ Difference, 95% CI	OCS Use OR, 95% CI	Resource Utilization OR, 95% CI
At Least One Allergen	−0.15 (−0.40, 0.09)	0.29 (0.01, 0.56) *	1.24 (0.75, 2.06)	0.54 (0.29, 0.99)
Cat Dander	−0.14 (−0.40, 0.11)	0.36 (0.08, 0.65) *	1.51 (0.91, 2.52)	0.66 (0.33, 1.31)
Dog Dander	0.04 (−0.28, 0.35)	0.21 (−0.15, 0.56)	0.83 (0.43, 1.60)	0.58 (0.23, 1.45)
Mouse Epithelium	0.12 (−0.21, 0.44)	0.04 (−0.32, 0.40)	0.39 (0.18, 0.86) *	0.62 (0.25, 1.54)
*Dermatophagoides Farinae*	0.03 (−0.23, 0.28)	0.10 (−0.18, 0.39)	1.06 (0.63, 1.77)	0.71 (0.36, 1.38)
*Dermatophagoides Pteronyssines*	−0.11 (−0.38, 0.15)	0.14 (−0.15, 0.44)	0.76 (0.44, 1.32)	0.75 (0.38, 1.48)
Cockroach	−0.04 (−0.37, 0.29)	0.01 (−0.35, 0.37)	0.91 (0.47, 1.77)	0.50 (0.19, 1.33)
*Alternaria Tenuis*	−0.33 (−0.73, 0.06)	0.39 (−0.06, 0.83)	0.55 (0.22, 1.38)	0.32 (0.07, 1.36)
*Aspergillus Fumigatus*	−0.63 (−1.17, −0.09) *	0.53 (−0.05, 1.12)	0.56 (0.18, 1.77)	0.29 (0.04, 2.25)

CI: confidence interval; OR: odds ratio; OCS: oral corticosteroids, ACQ: Asthma Control Questionnaire, AQLQ: Asthma Quality of Life Questionnaire. * Statistically significant at a <0.05 level.

**Table 4:** Adjusted associations between sensitization to indoor allergens and asthma morbidity in World Trade Center rescue and recovery workers.

Exposure	Mean ACQ Difference, 95% CI	Mean AQLQ Difference, 95% CI	OCS Use OR and 95% CI	Utilization OR and 95% CI
At least One Allergen	−0.19 (−0.43, 0.04)	0.27 (0.03, 0.50) *	1.16 (0.65, 2.07)	0.41 (0.20, 0.85) *
Cat Dander	−0.09 (−0.34, 0.16)	0.17 (−0.09, 0.43)	1.46 (0.80, 2.66)	0.47 (0.21, 1.06)
Dog Dander	−0.06 (−0.37, 0.24)	0.29 (−0.02, 0.61)	0.77 (0.37, 1.62)	0.45 (0.16, 1.27)
Mouse Epithelium	0.20 (−0.11, 0.50)	−0.08 (−0.39, 0.24)	0.42 (0.17, 1.02)	0.41 (0.13, 1.31)
*Dermatophagoides Farinae*	0.02 (−0.23, 0.27)	0.06 (−0.19, 0.31)	1.10 (0.60, 2.00)	0.48 (0.21, 1.08)
*Dermatophagoides Pteronyssines*	−0.24 (−0.49, 0.01)	0.29 (0.03, 0.55) *	0.77 (0.40, 1.46)	0.56 (0.24, 1.31)
Cockroach	−0.29 (−0.61, 0.03)	0.30 (−0.03, 0.63)	0.81 (0.36, 1.80)	0.27 (0.08, 0.91) *
*Alternaria Tenuis*	−0.10 (−0.48, 0.28)	0.09 (−0.30, 0.48)	0.49 (0.17, 1.38)	0.31 (0.06, 1.60)
*Aspergillus Fumigatus*	−0.42 (−0.94, 0.10)	0.46 (−0.08, 0.99)	0.42 (0.10, 1.69)	0.44 (0.05, 4.14)

CI: confidence interval; OR: odds ratio; OCS: oral corticosteroids, ACQ: Asthma Control Questionnaire, AQLQ: Asthma Quality of Life Questionnaire. * Statistically significant at a <0.05 level.
